# Cytokines in Pericardial Effusion of Patients with Inflammatory Pericardial Disease

**DOI:** 10.1155/2012/382082

**Published:** 2012-04-11

**Authors:** Konstantinos Karatolios, Rainer Moosdorf, Bernhard Maisch, Sabine Pankuweit

**Affiliations:** ^1^Department of Cardiology, University Hospital Gießen & Marburg, Baldinger Straße, 35043 Marburg, Germany; ^2^Department of Heart Surgery, University Hospital Gießen & Marburg, Baldinger Straße, 35043 Marburg, Germany

## Abstract

*Background*. The role of inflammatory and angiogenic cytokines in patients with inflammatory pericardial effusion still remains uncertain. *Methods*. We assessed pericardial and serum levels of VEGF, bFGF, IL-1*β* and TNF-*α* by ELISA in patients with inflammatory pericardial effusion (PE) of autoreactive (*n* = 22) and viral (*n* = 11) origin, and for control in pericardial fluid (PF) and serum (*n* = 26) of patients with coronary artery disease (CAD) undergoing coronary artery bypass graft surgery. *Results*. VEGF levels were significantly higher in patients with autoreactive and viral PE than in patients with CAD in both PE (*P* = 0, 006 for autoreactive and *P* < 0, 001 for viral PE) and serum (*P* < 0, 001 for autoreactive and *P* < 0, 001 for viral PE). Pericardial bFGF levels were higher compared to serum levels in patients with inflammatory PE and patients with CAD (*P* ≤ 0, 001 for CAD; *P* ≤ 0, 001 for autoreactive PE; *P* = 0, 005 for viral PE). Pericardial VEGF levels correlated positively with markers of pericardial inflammation, whereas pericardial bFGF levels showed a negative correlation. IL-1*β* and TNF-*α* were detectable only in few PE and serum samples. *Conclusions*. VEGF and bFGF levels in pericardial effusion are elevated in patients with inflammatory PE. It is thus possible that VEGF and bFGF participate in the pathogenesis of inflammatory pericardial disease.

## 1. Introduction

The management of pericardial effusion is an important clinical problem that remains challenging. However, the pathogenesis of pericardial fluid accumulation in different forms of pericardial disease is still not completely understood. Inflammatory reactions are a major cause of pericardial effusion. Studies on inflammatory pericardial effusions may elucidate the mechanisms of pericardial fluid formation and also the general mechanisms of pericardial inflammation. Cytokines are essential mediators of normal and pathologic immune and inflammatory responses. Particularly, inflammatory and angiogenic cytokines are involved in the pathogenesis of cardiovascular diseases, including atherosclerosis, myocardial infarction, myocarditis, and cardiomyopathy [1–3]. However, very few data exist on the activation pattern and pathophysiological role of inflammatory and angiogenic cytokines in pericardial diseases [4–6]. There are numerous studies on cytokines in pleural effusion [7–12] in contrast to the limited experience on pericardial cytokines in pericardial effusion. Cytokines, such as vascular endothelial growth factor (VEGF), basic fibroblast growth factor (bFGF), and interleukin-1*β* (IL-1*β*) and tumor necrosis factor-*α* (TNF-*α*) are candidates for the induction of pericardial effusions because they have been implicated in the induction and amplification of inflammatory reactions in exudative effusions [7, 9, 10, 13–17]. Therefore we measured the concentrations of VEGF, bFGF, IL-1*β*, and TNF-*α* in pericardial fluid and serum from patients with inflammatory, nonmalignant pericardial effusion and patients without pericardial disease.

## 2. Materials and Methods

### 2.1. Study Population

The study population included 33 consecutive patients with inflammatory pericardial effusion (PE) who underwent subxiphoid, fluoroscopically guided pericardiocentesis, pericardioscopy, and peri-/epicardial biopsy for therapeutic and/or diagnostic reasons after written informed consent [18]. For comparison, pericardial fluid (PF) from 26 patients was obtained and served during open-heart surgery for coronary artery disease together with a blood sample for each patient. None of the patients of the control group had a history of pericardial disease or evidence of pericardial effusion in echocardiography. The study was approved by the local ethics committee.

The etiologic diagnosis of inflammatory PE followed the criteria defined by the task force on pericardial diseases of the European Society of Cardiology [19]. In brief, the diagnosis of autoreactive inflammatory PE was based on the following criteria [19, 20]: (1) increased number of lymphocytes/mononuclear cells > 5000/mm^3^ or the presence of antimyocardial antibodies in pericardial fluid; (2) inflammation in epicardial biopsies by ≥14 cells/mm^2^; (3) exclusion of active viral infection in PE and epicardial biopsies (no virus isolation, negative PCR for major cardiotropic viruses, no IgM-titer against cardiotropic viruses in the PE); (4) exclusion of tuberculosis, Borrelia burgdorferi, Chlamydia pneumonia, and other bacterial infections by polymerase chain reaction (PCR) and/or cultures; (5) absent neoplastic infiltration in pericardial fluid and biopsy specimens; (6) exclusion of systemic, metabolic disorders and uremia. Viral inflammatory pericarditis was diagnosed by the presence of viral genome (parvovirus B19, influenza A/B, cytomegalovirus, enterovirus, adenovirus, herpes simplex virus, and Epstein Barr virus) detected by PCR in pericardial fluid and/or peri-/epicardial biopsies. For extraction of DNA/RNA from PE and peri-/epicardial biopsies, the QIAamp Blood Mini Kit and the QIAamp Tissue Kit (Qiagen, Hilden, Germany) were used. Conditions for PCR and primers have been described elsewhere [20].

### 2.2. Sampling of Pericardial Effusion, Pericardial Fluid, and Peri-/Epicardial Biopsies and Serum

In patients with inflammatory PE, pericardial effusion samples were obtained with pericardiocentesis. Pericardial fluid from patients with coronary artery disease (CAD) was obtained immediately after incision of the pericardium during coronary artery bypass graft surgery. Pericardiocentesis was performed in the cardiac catheterization laboratory using the subxiphoid route under local anaesthesia, with electrocardiographic and haemodynamic monitoring. After aspiration of pericardial fluid, a 0.038′′ J-tip guidewire was introduced and its position checked in the lateral 90 degrees, the posterior-anterior, and the right anterior oblique views. The guide wire was finally exchanged for a 7 French pigtail catheter. Pericardial fluid was removed by manual suction. Pericardioscopy was performed in the same session as pericardiocentesis. After the evacuation of the pericardial effusion, the pericardial catheter was exchanged for a 16 French introductory sheath. A flexible endoscope (Karl Storz AF 1101 Bl) was introduced in the pericardial space and up to eight peri-/epicardial biopsies were taken under direct eye control through the working channel of the instrument.

PE samples were divided for laboratory analysis including basic biochemical and cell count parameters, cytology, bacterial cultures, and polymerase chain reaction (PCR) for identification of cardiotropic viruses (influenza virus A/B, Parvovirus B19, cytomegalovirus, enterovirus, adenovirus, human herpes virus 6, and Epstein Barr virus), as well as Borrelia burgdorferi, Chlamydia pneumonia, and Mycobacterium tuberculosis. For cytokine measurement all PE, pericardial fluid (PF), and serum samples were promptly aliquoted, transferred into chilled sterile tubes containing a proteinase inhibitor cocktail (Complete; Roche, Penzberg, Germany), and subsequently stored at −80°C until analysis.

The epi- and pericardial biopsies were fixed and processed in the usual manner, embedded in paraffin, cut into 4 mm serial sections by microtome, and then stained with haematoxylin-eosin for routine histology and Ziehl-Neelson stain for mycobacteria. Pathohistology examination was always performed by two independent experts.

For immunochemistry, biopsy samples were separately fixed and processed for identification and characterization of infiltrating cells with monoclonal antibodies, bound immunoglobulins (IgP, IgG, IgM and IgA), and complement fixation. The following markers on infiltrating cells were analyzed: CD2, CD3, CD4, CD8, CD19, CD 16, CD45Ro, T200, CD54, CD25, CD14, and CD11c.

### 2.3. Immunoassay for VEGF, bFGF, IL-1*β*, and TNF-*α*

The levels of cytokines have been successfully determined in pleural and pericardial effusion in previous investigations using an enzyme-linked immunosorbent assay [4, 7, 16, 21, 22]. In the present study, cytokine levels in PE and serum were measured blinded to any clinical information with a commercially available sandwich enzyme-linked immunosorbent assay (ELISA) from R&D Systems (Quantikine colorimetric sandwich ELISA, R&D Systems) according to the manufacturer's guidelines. In brief, after standard procedures, for each cytokine all samples were pipetted into the wells of the microtitre plates, specific horseradish peroxidase-linked polyclonal antibodies were added, and immunoreactive levels of the cytokine were determined. The detection limit for VEGF_165_, bFGF, IL-1*β*, and TNF-*α* was 9 pg/mL, 3 pg/mL, 1 pg/mL, and 1,6 pg/mL, respectively. Both interassay and intra-assay coefficients of variation were <10% for VEGF_165_, bFGF, IL-1*β*, and TNF-*α*.

### 2.4. Statistical Analysis

Results are given as mean ± standard deviation. Values below the detection limit were assumed to be zero for statistical analysis. All *P* values < 0.05 were considered statistically significant. For statistical analysis the software packages SigmaPlot version 11.0 were used. Comparison of VEGF levels between the three groups was performed by Kruskal-Wallis test. In case of significant differences between the groups, closed testing principle was applied to compare two groups by use of the Mann-Whitney *U* test. Correlation between variables was calculated using the Spearman test.

## 3. Results

According to the results of pericardial effusion and peri-/epicardial biopsy samples analysis, 22 (66%) patients had autoreactive inflammatory PE (12 females, 10 males, 63,5 ± 11,6 years), and 11 (33%) patients had viral inflammatory PE (3 females, 8 males, 58,7 ± 11,7 years). For comparison, 26 patients with CAD (5 females, 21 males, 68,58 ± 8,3 years) were included in this investigation (Table 1).

VEGF was measurable in 10 (45%) PE and 18 (82%) blood samples from patients with autoreactive PE and in 9 (82%) PE and 8 (73%) blood samples from patients with viral PE. In CAD patients VEGF was detected only in 3 (12%) PF and 4 (15%) blood samples.

**Figure 1:**
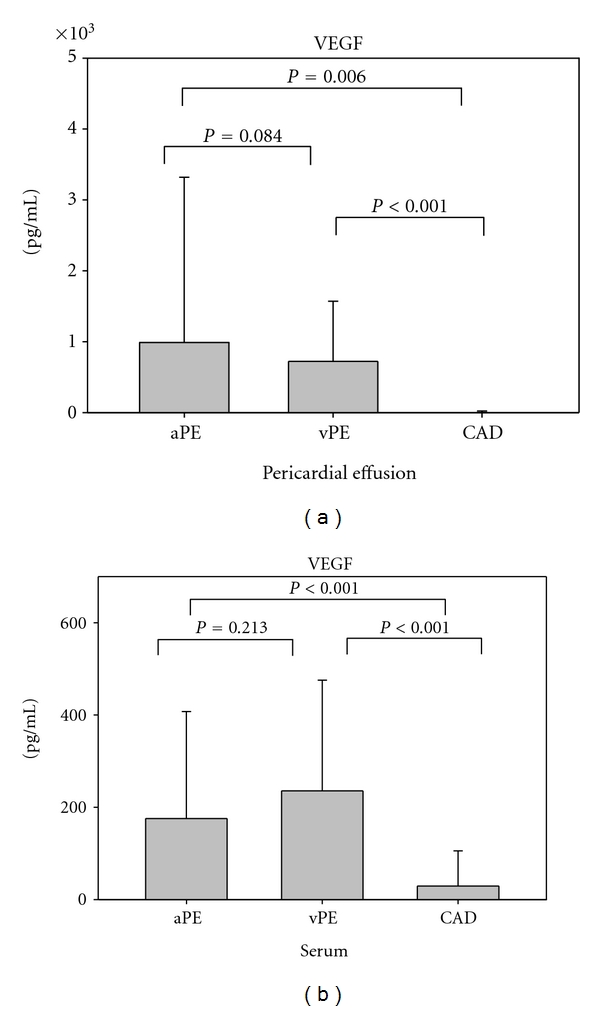
VEGF levels measured in pericardial effusion/pericardial fluid (a) and serum (b) from patients with autoreactive pericardial effusion, viral pericardial effusion, and coronary artery disease.aPE: autoreactive pericardial effusion; vPE: viral pericardial effusion; CAD: coronary artery disease.

Pericardial VEGF levels in autoreactive and viral pericardial effusion were significantly higher compared to VEGF in PF (*P* = 0,006 and *P* < 0,001, resp.) (Figure 1(a)). However, pericardial VEGF levels did not differ between autoreactive and viral PE (*P* = 0,084). In serum, VEGF was significantly higher in patients with autoreactive and viral PE compared to patients with CAD patients (*P* < 0,001 and *P* < 0,001, resp.). As in PE, serum VEGF levels did not differ significantly between patients with autoreactive and viral PE (*P* = 0,213) (Figure 1(b)). Although statistical significance was not reached, pericardial VEGF levels were higher than matched serum levels in 6 out of 22 (27%) patients with autoreactive PE and in 6 out of 11 (55%) patients with viral PE, (*P* = 0,07 for autoreactive PE; *P* = 0,279 for viral PE).

**Figure 2:**
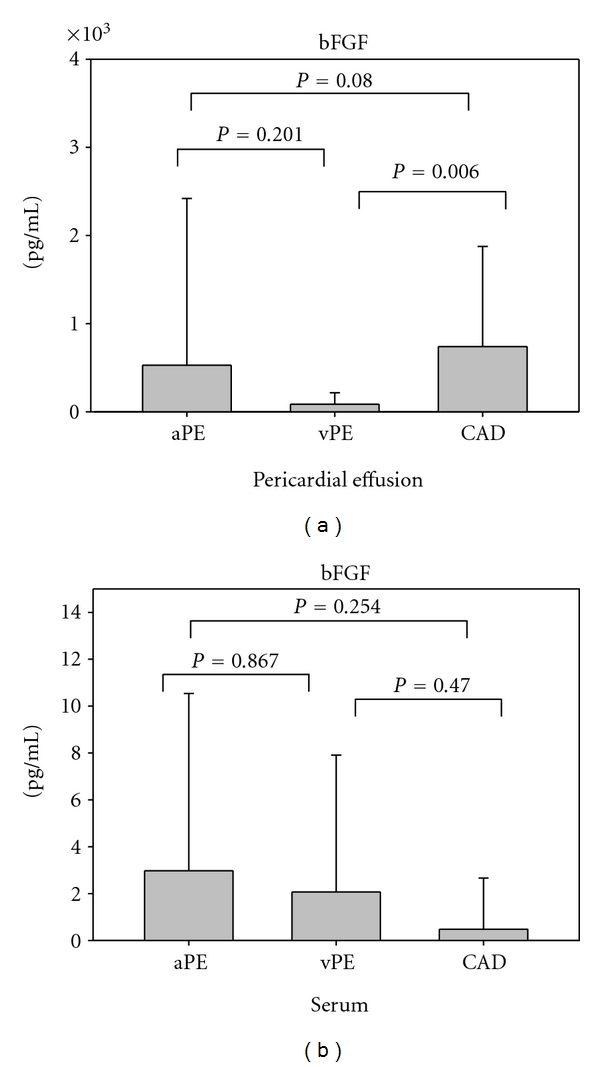
Basic FGF levels measured in pericardial effusion/pericardial fluid (a) and serum (b) from patients with autoreactive pericardial effusion, viral pericardial effusion, and coronary artery disease.aPE: autoreactive pericardial effusion; vPE: viral pericardial effusion; CAD: coronary artery disease.

**Figure 3:**
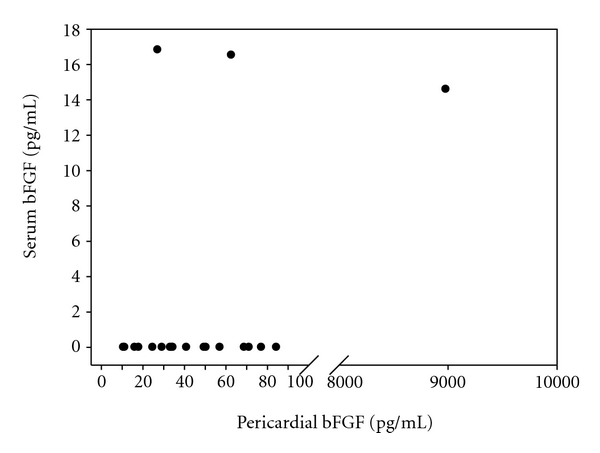
Pericardial and corresponding serum bFGF levels in patients with inflammatory pericardial effusion.

Basic FGF was detectable in 20 (91%) PE and 3 (14%) serum samples from patients with autoreactive PE, 10 (91%) PE samples and 1 (9%) serum sample from patients with viral PE, and 25 (96%) PF samples and 1 (3,8%) serum sample from patients with CAD. Pericardial bFGF levels were highest in patients with CAD, although statistical significance was only reached between CAD patients and patients with viral PE (*P* = 0,006) (Figure 2(a)). In serum, bFGF was detectable only in 3 patients with autoreactive PE, 1 patient with viral PE and 1 patient with CAD (Figure 2(b)). Pericardial bFGF levels were higher compared to serum levels in patients with CAD and patients with inflammatory PE (Figure 3) (*P* ≤0,001 for CAD; *P* ≤0,001 for autoreactive PE; *P* = 0,005 for viral PE).

IL-1*β* was detectable only in few PE (1 patient with viral PE and 1 patient with CAD) and serum samples (1 patient with viral PE). Obtained pericardial and serum levels did not differ between autoreactive PE, viral PE, and CAD.

TNF-*α* was detectable in PE of a smaller proportion of patients with inflammatory PE (2 patients with autoreactive PE and 3 patients with viral PE) and in none of the patients with CAD. In serum, none of the patients with inflammatory PE or CAD had detectable TNF-*α* levels.

Related biochemical parameters and leukocytes counts in PE from patients with autoreactive and viral PE are depicted in Table 2. Pericardial VEGF levels correlated positively with markers of pericardial inflammation (Table 3). In contrast, a negative correlation between pericardial bFGF and markers of pericardial inflammation was observed (Table 3).

## 4. Discussion

The present study investigated for the first time the pericardial and serum concentrations of VEGF, bFGF, IL-1*β*, and TNF-*α* in pericardial effusion and serum from patients with inflammatory pericardial effusion and compared them with pericardial fluid and serum levels of patients with CAD and no history or evidence of pericardial disease. Analysis of cytokines in pericardial effusion provides a modality to elucidate the pathophysiology of pericardial disease and may help to identify the etiology in patients with PE [19, 23]. However, studies related to cytokine analysis in pericardial effusion are rare. Initial investigations on pericardial cytokines included only case reports [5, 6]. In contrast, a systematic analysis of pericardial interleukin-6, interleukin-8, and interferon-*γ* investigated 101 patients with pericardial effusion [4]. However, intrapericardial VEGF, bFGF, IL-1*β*, and TNF-*α* levels in inflammatory PE have not been studied before.

In contrast to the limited experience on cytokines in inflammatory pericardial effusion, numerous investigations on cytokines in pleural effusion exist. Some of the findings could be possibly extrapolated for pericardial effusion.

Pleural (and pericardial) inflammation is typically accompanied by enhanced vascular permeability and both are essential for the pathogenesis of inflammatory effusions leading to increased fluid formation. Several studies confirm that VEGF, which has potent angiogenic, mitogenic, and permeability-inducing properties could be implicated in the pathogenesis of exudative pleural effusions. This notion is largely based on the findings that the levels of VEGF were consistently higher in exudative than in transudative pleural effusions [7, 16, 21, 24] and that pleural VEGF levels correlate with markers of pleural inflammation [16, 22]. In line with this data, we found higher pericardial VEGF levels in exudative, inflammatory PE than in PF from patients with CAD. Moreover, VEGF levels in inflammatory PE correlated positively with markers of pericardial inflammation (leukocytes and LDH in pericardial effusion).

A recent study by Wörnle et al. also demonstrated that viral infections lead to an increase in VEGF synthesis from mesothelial cells [12]. According to the authors mesothelial VEGF synthesis after activation of viral receptors could represent the link between viral infections and formation of pleural effusion. Further studies are warranted to investigate whether these findings also apply for viral pericardial effusion.

The implication of bFGF in the pathogenesis of exudative effusions has been less extensively studied. Data from Ruiz et al. [16] have suggested that bFGF participates in the pathogenesis of exudative pleural effusions and Strizzi et al. [25] have demonstrated a larger contribution of bFGF in benign than malignant effusions. However, the study by Ishimoto et al. [26] questioned the implication of bFGF in the formation of pleural effusion. In our series, pericardial bFGF levels were regularly elevated in patients with inflammatory PE.

Inflammatory cells and activated endothelium can synthesize and release bFGF [17, 27–29]. The inflammatory reaction itself may also cause endothelial cell damage, which results in increased bFGF production and release [30]. Basic FGF may amplify the inflammatory reaction by recruiting inflammatory cells [31, 32]. Furthermore, bFGF can induce vascular permeability directly and indirectly by upregulating VEGF [33]. However, bFGF was negatively correlated with markers of pericardial inflammation (leukocytes and LDH). One possible explanation may be that long-lasting exposure to bFGF reduces leukocyte migration and response to various chemokines [31, 34, 35] after the initial amplification of the inflammatory response. Further study is required to corroborate this finding.

In inflammatory pericardial effusion, the presence of cytokines within the pericardial cavity may be explained by intrapericardial production or diffusion from systemic circulation. Therefore, we compared pericardial and serum levels of the VEGF, bFGF, IL-1*β*, and TNF-*α* in order to examine whether local pericardial production is the main source of these cytokines. We observed that pericardial bFGF was significantly higher compared to corresponding serum bFGF, whereas pericardial VEGF tended to be higher than serum levels, but the difference did not reach statistic significance. These findings may reflect preferential local pericardial production of bFGF, whereas our results seem less clear for VEGF. Within the pericardial cavity, cytokines are likely to originate from multiple cellular sources. Infiltrating leukocytes, activated mesothelial cells, or epicardial adipose tissue may be involved. [17, 27, 28, 36]. The positive correlation between pericardial VEGF and pericardial leukocytes suggests that pericardial leukocytes might be one source of VEGF. Several investigations have also shown that pleural mesothelial cells are metabolically active and possess the capacity to produce various cytokines [37–39]. However, so far no study addresses the role and direct participation of pericardial mesothelial cells in the pathogenesis of pericardial inflammation.

In addition, we observed markedly elevated bFGF levels in PF from patients with CAD. In agreement with our results, high bFGF levels in PF of patients with ischemic heart disease have been reported before [1]. It has been suggested that bFGF in PF may accelerate the growth of human vascular smooth cells, induce angiogenesis, and mediate collateral development [1, 40]. Intrapericardial administration of bFGF promoted angiogenesis and reduced infarction size in dogs with acute myocardial infarction [41].

IL-1*β* and TNF-*α* were detectable only in a small proportion of patients with inflammatory PE. These results suggest that these cytokines play a minor part in inflammatory PE.

In conclusion, this study provides new data regarding the role of cytokines in the pathophysiology of inflammatory pericardial effusions. Elevated VEGF and bFGF levels in inflammatory pericardial effusions indicate that these growth factors might be involved in the pathogenesis of inflammatory pericardial effusions.

## Figures and Tables

**Table 1:** Demographic characteristics of study patients.

Variable	All patients	aPE	vPE	CAD	*P* value*
	(*n* = 59)	(*n* = 22)	(*n* = 11)	(*n* = 26)	
Men/women	39/20	10/12	8/3	21/5^#^	<0.05
Age (years)	66.7 ± 11.4	63.5 ± 11.6	58.7 ± 11.7	68.58 ± 8.3^‡^	<0.05
CAD	27 (46%)	1 (5%)	0	26 (100%)^#‡^	<0.001
Hypertension	42 (71%)	11 (50%)	5 (45%)	26 (100%)^#‡^	<0.001
Diabetes mellitus	12 (20%)	2 (4%)	0	10 (38%)^#‡^	<0.05
Hypercholesterolemia	44 (75%)	14 (64%)	6 (55%)	24 (92%)^#‡^	<0.05
Creatinine (mg/dL)	1.2 ± 1.24	0.88 ± 0.23	1.7 ± 2.5	1.29 ± 0.6^#^	<0.05
Hemoglobin (g/L)	135 ± 18.77	138.86 ± 15.6	124.36 ± 22.72	137 ± 18.21	NS
Leukocytes (G/L)	8.53 ± 2.81	8.62 ± 3.11	9.78 ± 2.99	7.61 ± 1.98	NS
Protein (g/dL)	69.86 ± 7.98	71.57 ± 6.68^‡^	63.18 ± 9.11	72.06 ± 6.63^‡^	<0.05
C-reactive protein (mg/L)	21.88 ± 46.61	24.76 ± 48.66	43.46 ± 66.13	3.25 ± 4.47	NS

Values are mean ± SD or numbers of patients (percentage).

*Analysis of variance/chi-square or Fischer exact test.

^#^ *P* < 0.05 versus aPE; ^‡^ *P* < 0.05 versus vPE.

In 9 patients with CAD and 1 patient with aPE no biochemical data were available.

aPE: autoreactive pericardial effusion; vPE: viral pericardial effusion; CAD: coronary artery disease; NS: not significant.

**Table 2:** Pericardial levels of related biochemical parameters in autoreactive and viral PE.

Variable	Autoreactive PE	Viral PE	*P* value*
LDH (U/L)	405.1 ± 761.35	902.36 ± 1008.77	0.08
Protein (g/dL)	37.05 ± 13,59	36.55 ± 10.9	0.92
Glucose (mg/dL)	95.5 ± 14.15	97.36 ± 21.73	0.77
Leukocytes (G/L)	1.47 ± 3.33	1.46 ± 1.24	0.15

Values are mean ± SD.

*Analysis of variance/*t*-test.

In 2 patients with autoreactive PE no biochemical data were available.

PE: pericardial effusion.

**Table 3:** Correlation between VEGF and bFGF and markers of pericardial inflammation in patients with inflammatory pericardial effusion.

Variable		VEGF	bFGF
LDH (U/L) in PE	*r *	0.655	−0.542
	*P*	<0.001	0.002
Leukocytes (G/L) in PE	*r *	0.543	−0.443
	*P*	0.003	0.02

VEGF: vascular endothelial growth factor; bFGF: basic fibroblast growth factor; PE: pericardial effusion; *r*, Spearman coefficient of correlation; *P*: *P*-value.
